# What Is the Simillimum?

**Published:** 1890-05

**Authors:** M. A. A. Wolff

**Affiliations:** Gainesville, Texas


### WHAT IS THE SIMILLIMUM?

(Continued from January and March numbers.)

M. A. A. Wolff, M. D., Gainesville, Texas.

The patient again wrote as follows:

"February 25th.—About ten days ago and fifteen days after menses began, I felt pain and soreness about the bladder, urinating very often, and this trouble has increased so that I am in bed. I suffer intense burning and throbbing just at the mouth and neck of the bladder, and there is enlargement of the little 'kernels' in the groin and about my waist. I feel as though a rope as large as my wrist had been bound around me and I cannot stand up straight nor take a deep breath or sneeze or cough for the pain, which just goes half-way from the middle in front round the left side to the middle of my back. My head aches in the right side with a clutching feeling, and there is an ache between my shoulders. I have noticed the amount and frequency of urine which I have passed in twelve hours through the night. I pass about half a teacupful of light-colored water with no sediment, only a milky look; and was up nine times, passing it freely till it stopped, when the pain would make me suffer very severely. Had fever all night. About eight years ago I strained myself reaching up to adjust a stove-pipe, and a bad cold set in about the same time, and I became very sick with cystitis. I passed clots of blood as large as the end of my little finger every fifteen minutes, almost having a spasm when they passed. I have always been very sensitive in the region of the bladder since, and have had three attacks, though not so severe as the first. I have taken Cantharis and Apis.-mel. I cannot bear the weight of the bedclothes or a hand to touch me, and suffer greatly in my back. I expect menses Thursday (27th), but have felt no ache from that quarter yet. I used the sponge after menses, and on the 9th, 10th, and 11th nights after removing the sponge it smelled badly; since then no odor, but my face has had several ugly pimples, mostly about chin."

When Mr. —— brought this report he stated that she had taken one of the powders given to have in readiness for menses (Mag.-phos.$^{200}$). So I told him that she must wait for the effect, and gave no other prescription.

February 26th I received the following report: "I have had fever all night and still have it. While my body is very hot, cold chills are creeping over me all night. Intensely thirsty. Only had to get up one time in the night, suffering a little less pain each time; had severe headache all night and morning. No portion of my body seems to be free from pain (little hair-like pains), and my body is so sore I can hardly move. I have eaten nothing since day before yesterday. I was very stupid and sleepy all day yesterday, many times falling into a restless sleep. The dog jumped up against the bed and frightened me, and I broke out into a profuse perspiration, and the fever stopped for half an hour."

To this her mother, who had written the report after her dictation, added:

"Doctor, Mrs. —— is a *very sick* woman and looks *very badly*, but she will not take any medicine, only what you sent her, for fear it will interfere with your remedies. I wished her to take Bryonia for her headache; please tell her if it will make a difference."

I answered to take nothing but what I prescribed, and sent Baptisia DMM, one powder.

I think it right to state here that after having had almost summer weather, the thermometer went down on the 25th P. M. from 60° to 40°, on the 26th to 20°, 27th P. M., to 16°.

On this day I was called to Mrs. ——'s bedside at five P. M. Her mother stated that the medicine (Bapt.) had worked well, and that three hours after she had taken it she felt all right, but now—as I saw—her condition was somewhat like the last report. Repeated Baptisia.

March 3d.—Mr. —— called in the office to tell me that his wife was up attending to her household affairs, and the menses, so far, had been all right without any trouble as usual.

